# The Inclusion of African-American Study Participants in Web-Based Research Studies: Viewpoint

**DOI:** 10.2196/jmir.5486

**Published:** 2016-06-22

**Authors:** Bekeela Watson, Dana H.Z Robinson, Laura Harker, Kimberly R. Jacob Arriola

**Affiliations:** ^1^ Emory University School of Medicine Atlanta Clinical and Translational Science Institute Emory University Atlanta, GA United States; ^2^ Rollins School of Public Health Department of Behavioral Sciences and Health Education Emory University Atlanta, GA United States

**Keywords:** Web-based interventions, African-Americans, social media, Internet, research techniques

## Abstract

The use of Web-based methods for research recruitment and intervention delivery has greatly increased as Internet usage continues to grow. These Internet-based strategies allow for researchers to quickly reach more people. African-Americans are underrepresented in health research studies. Due to this, African-Americans get less benefit from important research that could address the disproportionate health outcomes they face. Web-based research studies are one promising way to engage more African-Americans and build trust with the African-American community. With African-Americans’ increasing access to the Internet using mobile phones and other mobile phone technologies, we advocate for efforts to increase the representation of African-Americans in research studies by using the Internet as a recruitment tool and conclude with recommendations that support this goal.

## Introduction

African-Americans are commonly underrepresented in research studies [1-5]. A myriad of concerns are commonly cited when describing the reasons for this underrepresentation and why African-Americans are reluctant to participate in research studies [6-15]. Central to this lack of willingness to take part in research is an overarching sense of distrust that stems from a history of medical injustices [11,16,17]. Although the Tuskegee Syphilis Study is often cited as the most salient historical example of injustice in research pertaining to African-Americans, there are many other examples of medical racism in African-Americans’ collective consciousness [18-20]. Additional apprehensions that African-Americans tend to report are often accompanied by a lack of understanding related to the importance of research and the research process, economic challenges (transportation, employment) related to participation [21,22], and inadequate recruitment efforts made by study investigators [21-23]. Despite the challenges with research participation, increased morbidity and mortality related to chronic disease, predisposition to certain health conditions, and disproportionate impact of illness and varying health outcomes [24,25] necessitate concerted effort for engaging this population in health research. The research literature provides limited guidance in terms of effective strategies for using technology as a recruitment platform for African-Americans. However, such strategies are needed to better harness the benefits of technology and engage African-American participation in health research.

Existing studies have commonly recruited African-Americans via religious institutions and affiliations, community networks and organizations, and one-on-one interaction [26]. There are several specific strategies that have demonstrated effectiveness in the past. These strategies entail (1) direct face-to-face interaction with individuals and groups; (2) the necessity for flexibility with the manner in which participants are contacted (mobile phone, in-person, email); (3) provision of varying forms of information (booklet, handout, flier); (4) tangible participant incentives; and (5) an in-person reassurance of comfort with the research process inclusive of open sharing of information, opportunity for questions, and assertion of confidentiality [21]. However, when research necessitates large and diverse samples, many of these strategies are time consuming and often cost-prohibitive. Internet-based studies, in particular may require a different approach to recruitment, given that African-Americans’ underrepresentation in Web-based studies is well documented [21,27-32]. The purpose of this paper was to argue for enhanced efforts to include African-American participants in Internet-based research studies, despite the many challenges with doing so, and offer practical recommendations for effective recruitment strategies.

## Relevant Theoretical and Ethical Considerations

There are important theoretical and ethical considerations that drive decisions about, and describe the importance of, how to engage African-Americans in Internet-based research. Childers and Skinner developed equity theory as a useful framework for understanding the processes that maximize participation in survey research; and we believe that its application within a Web-based research context has far-reaching implications [33]. The authors argue that in a research study, the researcher must establish trust with the participant if the expectation is for the participant to comply with the protocol for the study. Equity theory proposes that when a researcher and participant interact, there is a comparison of the input from the participant and the gains as a result of that input [33]. Thus, an effective interaction (exchange) would be reflected if the participant feels that his or her input is equal to the outcome or gain. The subsequent reward (outcome) can be monetary, in terms of an incentive in exchange for participating, or psychological, reflecting a symmetry with the research topic and his or her values [33]. In light of studies finding greater distrust of medical research among African-Americans than other groups [34-36], there is greater responsibility on behalf of researchers to ensure that participants perceive an equitable exchange with their research participation. For example, the recruitment and screening process could be structured so that a study staff member personally explains the role (input) and benefits (ie, incentive) of participation in lieu of a completely automated study that requires no interaction with study staff for participation. An explanation of this sort provides participants with an opportunity for a personal cost–benefit analysis for research participation that ideally tips the scales in favor of participating.

In addition to equity theory, one can think about African-Americans’ involvement in Web-based research through a research ethics lens. Health researchers are trained to abide by 3 ethical principles: respect for persons, beneficence, and justice [37]. From an ethical perspective, all research participants should be treated as autonomous individuals who are able to make their own decisions, and persons with diminished autonomy are protected. Under the principle of beneficence, harm to participants is minimized, and possible benefits of the research are maximized. However, the principle of justice is especially relevant to the current topic because it requires equity among research subjects and equal distribution of benefits across populations. This principle lays foundation for claims that a proportionate number of African-Americans participating in health research is necessary so that results can be generalized to African-Americans, and their health can be impacted by medical advances. With an increasing volume of Internet-based research, the opportunity for participation among African-Americans has expanded giving rise to greater opportunity for study participation and subsequent benefit of health research [38-43].

## Internet Research Advantages

Using the Internet for health research has several main advantages. First, recruiting participants through the Internet allows for faster data collection at a lower cost than traditional in-person and email-based methods [44]. Participants can be screened almost instantly, and information can be entered directly into Web-based databases, thus reducing the time-intensive manual data entry process. Second, Web-based recruitment has the potential for farther reach unlike traditional in-person recruitment, which is bound by geographic restrictions [45]. This is especially beneficial when the target population is relatively small, homebound, or lacks transportation and when studying a sensitive topic [14]. Given that 87% of people in the United States are estimated to have access to the Internet, the Internet can be a powerful tool for reaching these groups [46]. Internet use among young, college-educated people with higher incomes is comparable regardless of race and across Internet platforms, African-Americans and whites are very similar with mobile Internet access [47]. Mobile phones play a significant role bridging this Internet access gap [48]. Third, Web-based methods can eliminate common participant barriers to participation, such as lack of time, transportation, and scheduling [25]. Participating in research through the Internet tends to be more convenient as the participant can usually complete the research within his or her own time frame and location of preference [49].

Social media is one Web-based method that can be used to recruit specific individuals based on a specific demographic profile and ensure access to a diverse group of people [29]. For example, a 2014 study used social media sites to recruit HIV-positive individuals. Overall, 1221/1404 (87%) eligible participants completed the survey, indicating that Web-based recruitment was effective [49]. Using self-reported information on social media user profiles, researchers can target advertisements for specific populations and change content. For example, in a study conducted by Sullivan et al. participants were more likely to respond if the people pictured in the advertisements belonged to their racial or ethnic group [29].

Social media sites are also increasingly more popular among African-Americans. Research studies that use social media sites can especially benefit from recruiting a younger African-American audience, as this population uses social media at rates higher than comparable figures for whites [47]. Twitter specifically has a larger proportion of African-American users than whites based on results from a Pew Research study using a nationally representative sample (117/532, 22% of African-Americans; 579/3617, 16% of whites) [47]. Research has demonstrated that targeted ads on social media sites are effective at recruiting specialized populations [44,50]. For example, a 2013 Web-based preventive depression study recruited participants using search engine advertising (Google, Yahoo!, Bing), Facebook advertising, posts in forums and Web-based noticeboards, and promotion through relevant websites and email newsletters of mental health organizations. Several of the methods were effective, but Google Ads yielded the most participants [45]. Increasingly, researchers have used a variety of Web-based methods to recruit a broad sample of participants such as Web-based advertisements, Craigslist, and mobile phone apps [51].

## Internet Research Disadvantages

Although the digital divide has been closing over time and African-Americans represent a group adopting broadband Internet at rates greater than other ethnic groups, there are still gaps in Internet access in the United States [52]. Large disparities exist with regard to the location, frequency, duration, and type of Internet access; however, this gap is not consistent across technology platforms [47]. As of 2014, using a nationally representative sample, it was estimated that 531/664 (80%) of African-Americans had Internet access (compared with 3674/4223, 87% of whites), and 412/664 (62%) had a home broadband connection (compared with 3125/4223, 74% of whites) [47]. Internet usage rates are most notable when comparing differences between African-Americans (299/664, 45%) and whites (2660/4223, 63%) with respect to older adults and those with lower levels of education [47]. Consequently, persons with less access to technology use email and social networking apps less often than those with more consistent access. These differences can affect completion rates for Internet surveys and are likely confounded by education and literacy [26]. The differences in completion rates can then create bias within the sample, which affects the generalizability of study findings.

General challenges with Web-based research participation are also abundant and entail issues related to recruitment, response rate, study retention, generalizability, and overall study design [14,15]. Some common reasons for low response rates among potential participants include ignoring study emails or ads, lacking the motivation (e.g., incentives, personal connection to the study) to participate, and/or difficulty with distinguishing legitimate messages from spam [53]. Minority recruitment can be even more complicated and difficult when compared with recruitment of other populations because of the inherent characteristics of the Internet such as concerns with absolute authenticity, the necessity of flexibility with multiple recruitment strategies, and dependency on cooperation with gatekeepers [15]. These are all challenges that should be accounted for when considering the Internet as a tool for research studies targeting African-Americans.

## Recommendations

There is value in conducting Internet-based research studies targeting African-Americans to improve their health and well-being. Oftentimes, research with African-Americans requires a level of sensitivity that recognizes the influence of culture, history, and familial interactions. As a result, more efforts must be made by researchers who intend to include African-Americans in ways that align with equity theory [33].

We offer 5 recommendations to increase the successful recruitment of African-Americans into Internet-based research. First, consider using recruiters. In this approach, African-Americans are trained (via webinar) to recruit from their social networks both through the Internet and in person. The training should involve a brief overview of the reasons for African-Americans’ hesitancy to be involved in research studies (as we described previously), the importance of justice and equity theory as it relates to African-American recruitment, background information on the study, and techniques for Web-based recruitment such as social media. By using social media, trust is already established between the researcher (via the recruiter) and potential participants, increasing the likelihood of enrolling participants into the study. In addition to the potential for a large reach, social media recruitment has been demonstrated to be cost-effective when compared with traditional recruitment methods [43,54,55]. Second, and related to the first, is to capitalize from a commonly used technique—snowball sampling—in which participants recruit other participants [56]. Respondent-driven sampling is a commonly used form of snowball sampling [57]. These methods are being refined for use in a Web-based environment but may be particularly helpful for recruiting African-Americans because of previously established trusted relationships that exist among participants and members of their social networks.

Third, consider offering a list of incentives from which participants can choose. Each incentive must have the ability to be delivered quickly and with minimal effort through the Internet (eg, electronic methods for cash payment such as PayPal, electronic gift cards such as Amazon e-gift cards, and virtual debit or credit cards). An incentive that is worthwhile to the participant will cause him or her to feel that the exchange is equitable. That is, the participant perceives that he or she is receiving a reward equal to his or her effort. Fourth, 2-tiered recruitment is suggested when feasible (eg, there is a small geographic recruitment area). In this approach, participant recruitment occurs initially in person affording the participant an opportunity to interact with a trained peer volunteer and establish a rapport. Simultaneously, the participant will receive Web-based communication with directions for the completion of an Internet-based screener. The second tier of this approach entails the participant completing the screener, intervention components, and all questionnaires online. Decreased reach is a limitation of this approach because a researcher working face to face with participants cannot engage as many participants as he or she could do through the Internet. A final recommendation is to use trusted institutions as partners for participant recruitment. Institutions such as churches, community organizations, sororities, and fraternities have existing relationships with the communities of which they are a part. The investigator can establish relationships with members of the institutions directly then work with the institution to send emails to their membership regarding study participation.

## Conclusions

The convenient, far-reaching, fast-paced, Web-based environment provides numerous methodological advantages for researchers, yet specialized efforts are necessary to ensure the inclusion of African-American participants in Internet-based research studies. To build trust within African-American communities, address the unique challenges posed by participation in research. The hesitancy related to participation, is often a combination of a lack of understanding related to the relevancy of research [21,22] inadequate recruitment efforts made by study investigators [21-23], and a general lack of trust with respect to the health and medical field [11,16]. This viewpoint recommended 5 approaches that entail using recruiters and previous participants themselves, multiple-choice incentives, a 2-tiered recruitment strategy, and partnership with trusted organizations. These approaches emphasize the importance of relationship building as this has been highlighted as a key component of research recruitment and retention of African-American participants [21,58]. The use of these approaches may not be particular to African-Americans; however, they may be particularly useful for investigators to overcome some of the challenges related to mistrust and serve as a mechanism to establish a greater rapport with African-American research participants.
